# Interpersonal value profiles and analysis of adolescent academic performance and social thinking

**DOI:** 10.3389/fpsyg.2015.00575

**Published:** 2015-05-05

**Authors:** José J. Gázquez, Jorge Sainz, María del C. Pérez-Fuentes, María del M. Molero, Francisco J. Soler

**Affiliations:** ^1^Department of Psychology, Faculty of Educational Sciences and Health, University of AlmeríaAlmería, Spain; ^2^Department of Economics, Faculty of Law and Social Sciences, University Juan Carlos IMadrid, Spain

**Keywords:** interpersonal values, academic performance, social competence, social thinking

## Abstract

The purposes of this study were to identify interpersonal value profiles and find out whether there were any differences in academic performance and social thinking. The study sample was 885 high school students of whom 49.8% (*N* = 441) were boys and 50.2% (*N* = 444) were girls. The results show that students with low Benevolence and Conformity levels showed higher prevalence of failures and repeated the year more often. Furthermore, students with a high level of Recognition and Leadership and low Conformity and Benevolence are socially incompetent students. Intervention programs should to achieve high levels of kindness and consideration, respect for rules and generosity, and diminish the perception of recognition by others and exertion of authority. Thus, this study shows the values that must be worked on to improve students’ Academic Performance and social competence.

## Introduction

### Concept and Acquisition of Interpersonal Values

Interpersonal values are convictions about a certain behavior model the individual has at a given time, and which is personally or in the view of society, is preferable to another mode of behavior (Kornblit, 2003). These interpersonal values have awakened the attention of research in the Social Sciences (Pertegal et al., 2010), where they are analyzed for the function they fulfill in decision-making (Wallace et al., 2006), avoiding aggressive behavior (Benson et al., 2006), and finding out what values make a subject socially competent (Oliva et al., 2010).

Values are acquired in the family and educational environments, where the family provides the first experience for beginning construction of the individual’s identity and facilitates acquisition of a primary value structure (Fuentes et al., 2011), while in the education system, youths can interact with others and establish a hierarchical classification of the value system acquired (Jiménez et al., 2008).

In adolescence, the family context continues to influence shaping of the psychosocial connection, even though peer group contributions seem to be determinant. Results of some studies show that an adequate positive environment exerts a favorable effect on the development of the adolescent and his/her behavior (Zimmer-Gembeck and Locke, 2007). For example, behavior problems in adolescents are often the result of a family environment where there is a lack of affectivity (de la Torre et al., 2013). In the opinion of the family members themselves, a weak education in values is associated with problems living together.

Values are more abstract in women than in men, who give them a more egocentric and instrumental load. As they grow older, adolescents show preference for values more in harmony with terms of equality and dignity, and start opposing more egocentric values centered around “I,” or confrontation with others (Martí and Palma, 2010).

### Academic Performance and Interpersonal Values

Since the 20th century, academic performance, specifically during the period of education before university, has been transformed into one of the major problems of industrialized cities, attracting the attention of both students and teachers (Abalde et al., 2009). Around 50 years ago, it began to be shown that academic performance, in addition to depending on individual components, was also influenced by sociocultural agents, such as sex, parents’ occupation and education, values and attitudes toward education, etc., (Cú and Aragón, 2006).

In general, the school is an institution where children should be provided with all the resources necessary to become successfully integrated in their society (López et al., 2002), and confer them with better academic performance (Garaigordobil, 2005; Inglés et al., 2013). Very few studies have been done on this (Martín et al., 2008), although it is clear that both violent behavior and academic failure are two of the problematic situations we come up against in high schools (Gázquez et al., 2010).

Garner (1988) suggested that just as other cognitive agents, orientation in values students have about learning is a differentiating agent in academic performance. Inglés et al. (2013) relate academic performance to a lack of values. It has also been found that bullies (Cerezo, 2001), and those who have a hard time integrating with other members of the group have lower academic performance, whereas the contrary is true of those who relate successfully (Walters and Bowen, 1997). Ros et al. (1996) did a study in which benevolence was associated favorably with study habits, somewhat like conformity, which showed the highest correlations with all study behaviors. On the contrary, power is negatively associated with study behavior, as is stimulation with study routines and planning. Therefore, one of our hypotheses is that students who show high benevolence and conformity and low stimulation have better academic performance.

### Cognitive-Social Strategies and Attitudes and Their Relationship to Interpersonal Values

Cognitive social strategies include skills, such as anticipation or observation, which the individual can use to confront the demands of the society he is immersed in (Diener and Kim, 2004). Gagné (1971) believes that the individual’s cognitive strategies are tactics that enable one to deal properly with information, reinforcing it and facilitating its retrieval to face any problems that arise. Along this same line, Mahar and Sullivan (2002) suggest that cognitive-social strategies give the individual an opportunity for successful social integration, and emphasize the usefulness of some of them for observing and retaining information, looking ahead to see the consequences of different types of behavior, problem-solving, etc. The attitude concept refers to mental and neurophysiological willingness, which is the product of experience and influences the subject and his reactions to objects and situations (Allport, 1935). These strategies, used in social relations along with the various cognitive attitudes, make up what is called social competence (Trianes et al., 1997; Nacimiento and Mora-Merchan, 2014).

Rokeach (1968a,b) suggests that values, attitudes, and behaviors are structured within a system of beliefs that rotates around the medulla of the social individual. It is not new to psychology that values are transcendental elements of personality which exercise a determining influence on people’s actions, and adolescence is essential for their development (Damon, 2004). One example, of how attitudes connect with interpersonal values is people who are the victims of violence, and who as a consequence, shape an unfavorable perception and negative values about their context, perceive it to be unsafe and threatening (Sutton and Smith, 1999), which simultaneously makes the person timid (Polo et al., 2013), introverted (Oñate and Piñuel, 2005), socially isolated (Moreno et al., 2006), etc. Concerning values and strategies, preferential strategies may sometimes be carried out, depending on the expectations of each individual and how he evaluates the situation, and on the contrary, in other situations this is not an option (Sanna et al., 1999).

Several studies have approached the association of values, attitudes, cognitive-social strategies, and the various ways in which they interrelate. Enciso and Lozano (2011) did a study with young people from 11 to 18 years of age who were in volunteer programs and others who were not, and arrived at the conclusion that the volunteer group showed signs of greater conformity with what was socially correct, helping and collaboration, prosocial leadership, etc. Cognitive strategies for solving social problems that stand out for their ability to observe the situation and retain information from it are finding alternative solutions, anticipating effects or consequences of certain actions, and the ability to select the best means to the goals pursued (Moraleda et al., 1998). A study by Castaño and León del Barco (2010) with 162 university students found that individuals who considered themselves extroverted and sociable made more use of individual strategies for contact with other students to whom they could tell their problems and difficulties, while those who considered themselves introverted, showed signs of strategies that distanced them from contact with others.

Arán and Richaud de Minzi (2011) found that children in socially vulnerable environments showed more cognitive impulsiveness than children who were at no social risk, which could be explained by the negligible cognitive stimulation which is representative of impoverished environments. Impulsiveness is also associated with childhood abuse (Fernández-Millán et al., 2002). Fossati et al. (2012) found kindness negatively associated with aggressive action, and low levels of kindness related to aggressive behavior, while high levels of kindness were with prosocial behavior. Thus we formulate another of our hypotheses relating benevolence, a value equivalent to kindness, to the presence of either aggressive or prosocial behavior, suggesting that less benevolence is related to aggressive behavior, while high levels of benevolence, on the contrary, are related to a subject’s prosocial behavior.

Isolation or social withdrawal is also directly associated with attention problems (Cluver and Gardner, 2006; Cao and Su, 2007). Finally, Cardozo et al. (2011), in a study with 124 students from 13 to 18 years old, found that those who scored highest in consideration of others also showed high levels of leadership and altruism, while those who scored low in consideration of others were more withdrawn and aggressive.

In view of the above, the final purpose of our work was to analyze the presence of differences in interpersonal value profiles on the social attitudes of the students, and for this we planned a series of specific goals: form groups characterized by different levels of the five interpersonal values, compare the differences among these groups with the academic performance of the students, and finally, find out to what extent these profiles show different values in the social thinking of high school students.

## Materials and Methods

### Participants

The sample was taken by random cluster sampling (Inglés et al., 2014), by geographic areas in the province of Almería Center, Levante (Eastside), and Poniente (Westside). It should also be mentioned that a sample of over 200 students was taken from each zone in the province [Center 212 students (24%), Levante 333 students (37.6%), and Poniente 340 students (38.4%)], and at least four classes in each school selected (two in third year two in fourth year).

A total of 1,055 students in third and fourth year of high school were included in the sample, of whom 120 students (11.37%) who were not native Spaniards were disqualified because they could not understand the Spanish language well enough and did not finish the questionnaire in time, and another 50 students (4.74%) were disqualified because of errors or omissions, or not having attended one of the two sessions it was given in. The final sample was made up of 885 high school students of whom 49.8% (*N* = 441) were boys and 50.2% (*N* = 444) were girls, in an age range of 14–18 with a mean of 15.2 years (SD = 0.90), for the total sample, and 15.22 (SD = 0.92) for boys and 15.19 (SD = 0.89) for girls.

### Instruments

#### Survey of Interpersonal Values (SIV; Gordon, 2007)

Based on 90 items with two answer choices (YES–NO), the test measures six areas of students’ relationships with others: Stimulation (Being treated kindly, considerately, understandingly, and perceiving support from others), Conformity (following norms, doing what is socially correct, conforming and acting according to what is accepted and suitable), Recognition (Being recognized by others, admired and thought well of, attract positive attention), Independence (Doing and considering that it is your right to do whatever you want, deciding for yourself with your own criteria and being free), Benevolence (Being generous, helping others, and doing and sharing things with them), Leadership (Exert authority over the people under you in the performance of a position of command or power). The Cronbach’s alpha is from 0.78 to 0.89 (Gordon, 1993).

#### Cuestionario de Actitudes and Estrategias Cognitivo Social [Cognitive-Social Attitudes and Strategies Questionnaire] (AECS)

It is developed by Moraleda et al. (1998) proposes a two-way factor structure of social thinking: at the positive pole are processes and strategies facilitating social relations and those that make adolescents socially competent, while at the negative pole are the processes and strategies that inhibit social relations, and which therefore make the adolescent socially incompetent. Each factor in turn is made up of subfactors, which measure different aspects of social thinking: Inhibitors (Positive perception of how parents exert authority in the home) and Facilitators (Convergence vs. divergence, Impulsiveness vs. reflectiveness, Field independence/dependence, negative perception and expectations about social relations, subject’s negative perception of the quality of acceptance and protection by parents, difficulty in observing and retaining relevant information about social situations, difficulty in finding alternative solutions to social problems, difficulty in anticipating and understanding the consequences that could follow social behaviors, difficulty in choosing adequate means to the ends pursued by social behavior). It uses a 7-point Likert-type scale to express extent of agreement with the statement. The Cronbach’s alphas were: for positive perception of how parents exert authority in the home (α = 0.67), Convergence vs. divergence (α = 0.46), Impulsiveness vs. reflectiveness (α = 0.69), Field independence/dependence (α = 0.56), negative perception and expectations about social relations (α = 0.56), subject’s negative perception of the quality of acceptance and protection by parents (α = 0.77), difficulty in observing and retaining relevant information about social situations (α = 0.75), difficulty in finding alternative solutions for solving social problems (α = 0.65), difficulty in anticipating and understanding the consequences that could follow social behavior (α = 0.71), difficulty in choosing adequate means to the ends pursued by social behavior (α = 0.75).

#### Academic Performance

Measured as a function of the items “Have you ever failed a subject?” and “Have you ever had to repeat a year?” with yes and no answer choices in both cases.

### Procedure

First, meetings were held with the directors and counselors at the schools selected to explain the purpose of our research, show them the instruments to be used and request their permission and cooperation to implement the study. After the parents had been informed in a meeting at which the researchers responsible were present, and their consent for participation by their children had been acquired, questionnaire administration was scheduled. The questionnaires were given in two 50-min sessions, with a rest period of variable length between them, separated by a class and a recreation period, or just recreation, but always over 20 min. The questionnaires were given in groups, voluntarily and anonymously in the classroom itself, or someplace else in the school if several classes took it together.

#### Statistical Analyses

Since it was a large sample, quick cluster analysis (Hair et al., 1998; Rodríguez et al., 2014) was used to identify interpersonal value profiles, that is, to classify the students in uniform groups. Profiles were defined from the combinations of the five Interpersonal Value factors evaluated by the SIV (Gordon, 2007), and inter-cluster differences were maximized by the number of clusters selection criterion. Furthermore, each of the groups that showed different interpersonal value profiles was theoretically feasible and psychologically meaningful.

When the groups had been formed, student distribution in the different groups by the failing and repeating variables was found using the Chi square test.

Apart from this, an analysis of variance (ANOVA) was done to find out the differences in social thinking among the groups, and the magnitude or effect size was found using eta square (η^2^). Where the differences were statistically significant, Scheffe *post hoc* tests were done to find out which groups they occurred between, because this test does not require equal sample sizes, and in our case, each group was made up of a different number of students. To calculate the magnitude of the differences observed, whenever there were any, the effect size, that is, the standardized mean difference or Cohen’s *d* (Cohen, 1988), was calculated. This was interpreted as *d* ≤ 0.50 being a small effect size, *d* ≤ 0.79 a medium effect size, and when *d* ≥ 0.80, it was a large effect. Statistical analyses were done with the SPSS 20 statistical package.

### Results

#### Identification of Interpersonal Value Profiles

**FIGURE 1:**
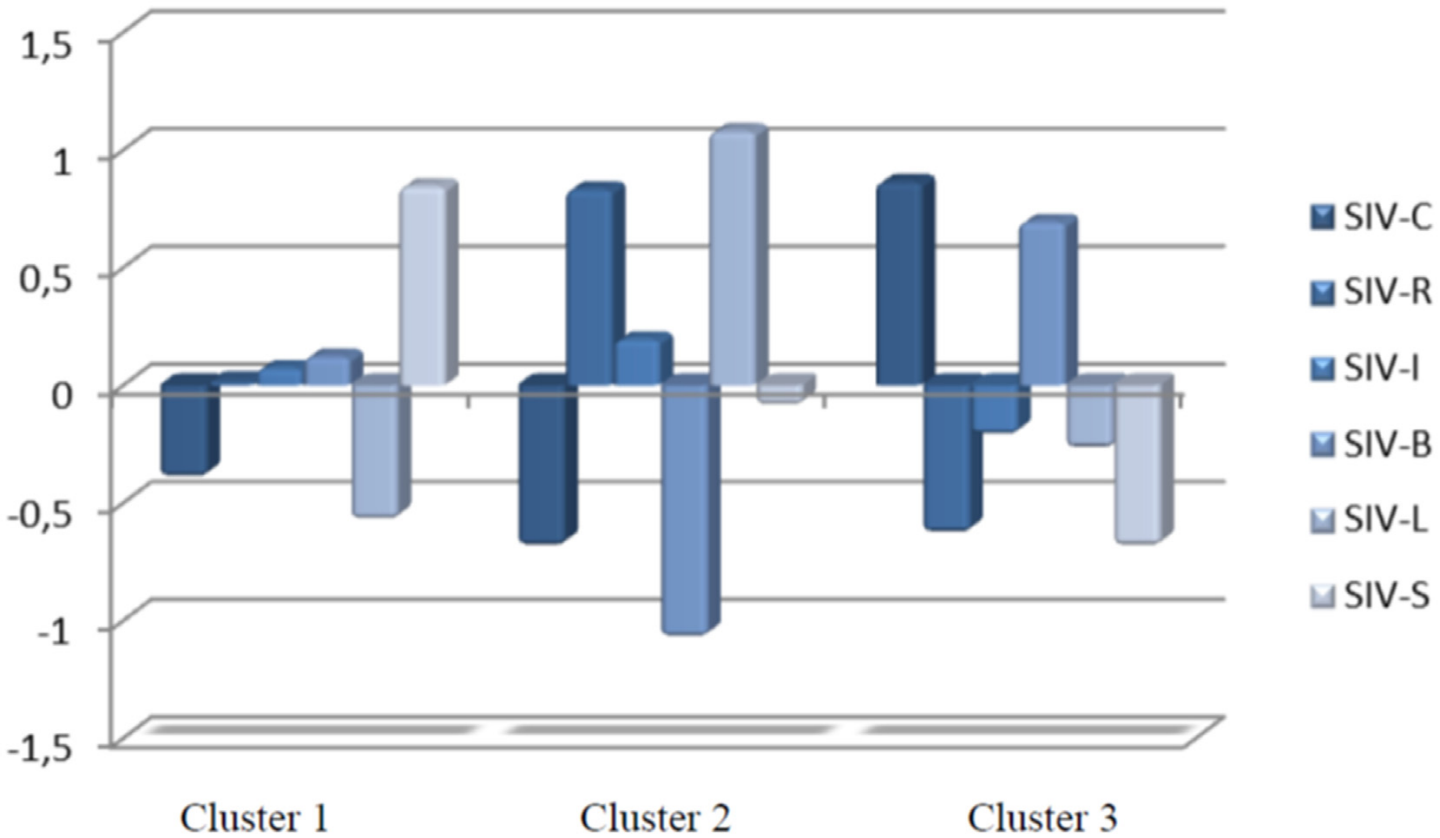
**Representation of the three-cluster model: Cluster 1 (*HS-LCL*), Cluster 2 (*HRL-LCB*), and Cluster 3 (*HCB-RSLI***). SIV-S, Stimulation; SIV-C, Conformity; SIV-R, Recognition; SIV-I, Independence; SIV-B, BenevoIence; SIV-L, Leadership.

In the cluster analysis it was attempted to make each group as homogeneous as possible and the intergroup differences as large as possible, while also considering their theoretical fit. The results enabled three groups to be differentiated by level of the five interpersonal values analyzed (**Figure 1**). This three-cluster solution was chosen because it emphasizes the role of Stimulation, Recognition, Benevolence and Leadership. These groups are:

1. Cluster 1 (*HS-LCL*): 288 students (32.5% of the sample) characterized by high Stimulation and low Leadership and Conformity
2. Cluster 2 (*HRL-LCB*): 236 students (26.7% of the sample) who had high levels of Recognition and Leadership and low Conformity and Benevolence
3. Cluster 3 (*HCB-RSLI*): Comprised of 323 students (36.5% of the sample) who had high Conformity and Benevolence and low Stimulation and Recognition.

### Intergroup Differences in Academic Performance

**Table 1** shows the distribution of three clusters or groups by whether they had failed or not and repeated or not. It may be seen how Group *HRL-LCB* (high Recognition and Leadership and low Conformity and Benevolence) shows statistically higher levels of students who failed (χ^2^ = 13.1, *p* = 0.01) and students who had repeated a year (χ^2^ = 12.44, *p* = 0.01).

**Table 1:** Profiles and prevalence of failing and repeating.

			HS-LCL (G_1_)	HRL-LCB (G_2_)	HCB-RSLI (G_3_)
Failed	NO	Count	65	25	61
		%	22.6%	10.6%	18.9%
	YES	Count	223	211	262
		%	77.4%	89.4%	81.1%
Repeated	NO	Count	217	144	223
		%	75.3%	61.0%	69.0%
	YES	Count	71	92	100
		%	24.7%	39.0%	31.0%

*HS-LCL (G_1_), high Stimulation-low Leadership and Conformity profile; HRL-LCB (G_2_), high Recognition and Leadership-low Conformity and Benevolence profile; HCB-RSLI (G_3_), high Conformity and Benevolence-low Stimulation and Recognition profile.*

That is, the highest percentages for both the failing and repeating variables are in the HRL-LCB Group with 89.4 and 39%, respectively. While the lowest prevalence is in the HS-LCL (high Stimulation and low Leadership and Conformity) Group with 77.4% of students who had failed and 24.7% who had repeated. Finally, the HCB-RSLI (high Conformity and Benevolence and low Stimulation and Recognition) Group has intermediate percentages of prevalence of students who failed (81.1%) and repeated (31%).

### Intergroup Differences in Social Thinking

As seen in **Table 2**, when the different Social Thinking scales are examined, the only scale in which there are no statistically significant differences by cluster or group is “*Positive perception of how parents exert authority in the home.*” The rest of the scales did show significant differences in mean scores, where Group HRL-LCB, tended toward inhibiting cognitive processes and strategies in social relations, with effect sizes varying from *d* = 0.21 to *d* = 0.43, which are both small.

**Table 2:** Mean and SD found for the three groups, η^2^ and Scheffe test for each Social Thinking scale.

Social thinking	Cluster	*N*	*M*	DT	*F*	*p*	η^2^	Scheffe	*d*
Cv	HS-LCL (G_1_)	288	18.55	4.65	6.18	0.01	0.01	| G_1_–G_2_|^∗^	0.22
	HRL-LCB (G_2_)	236	19.75	4.44				| G_2_–G_3_|^∗∗^	0.21
	HCB-RSLI (G_3_)	323	18.46	4.81				| G_1_–G_3_|	n.s.
Imp	HS-LCL (G_1_)	288	23.83	6.97	19.79	0.00	0.05	| G_1_–G_2_|^∗∗^	0.39
	HRL-LCB (G_2_)	236	26.69	6.58				| G_2_–G_3_|^∗∗^	0.38
	HCB-RSLI (G_3_)	323	23.02	7.32				| G_1_–G_3_|	n.s.
Ind	HS-LCL (G_1_)	288	21.69	5.82	22.74	0.00	0.05	| G_1_–G_2_|^∗^	0.42
	HRL-LCB (G_2_)	236	23.05	5.36				| G_2_–G_3_|^∗∗^	0.41
	HCB-RSLI (G_3_)	323	19.73	6.23				| G_1_–G_3_|^∗∗^	0.39
Dem	HS-LCL (G_1_)	288	21.20	6.32	1.97	0.14	n.s.	| G_1_–G_2_|	n.s.
	HRL-LCB (G_2_)	236	20.61	5.68				| G_2_–G_3_|	n.s.
	HCB-RSLI (G_3_)	323	21.63	5.94				| G_1_–G_3_|	n.s.
Per	HS-LCL (G_1_)	288	19.82	5.98	13.69	0.00	0.03	| G_1_–G_2_|^∗∗^	0.33
	HRL-LCB (G_2_)	236	21.85	5.82				| G_2_–G_3_|^∗∗^	0.32
	HCB-RSLI (G_3_)	323	19.25	6.07				| G_1_–G_3_|	n.s.
Hos	HS-LCL (G_1_)	288	14.45	6.79	16.38	0.00	0.04	| G_1_–G_2_|^∗∗^	0.36
	HRL-LCB (G_2_)	236	16.53	6.45				| G_2_–G_3_|^∗∗^	0.35
	HCB-RSLI (G_3_)	323	13.37	6.18				| G_1_–G_3_|	n.s.
Obs	HS-LCL (G_1_)	288	25.75	7.8	23.51	0.00	0.05	| G_1_–G_2_|^∗∗^	0.43
	HRL-LCB (G_2_)	236	29.33	7.37				| G_2_–G_3_|^∗∗^	0.42
	HCB-RSLI (G_3_)	323	24.90	8.14				| G_1_–G_3_|	n.s.
Alt	HS-LCL (G_1_)	288	26.64	6.73	15.93	0.00	0.04	| G_1_–G_2_|^∗∗^	0.35
	HRL-LCB (G_2_)	236	29.55	7.28				| G_2_–G_3_|^∗∗^	0.34
	HCB-RSLI (G_3_)	323	26.46	6.96				| G_1_–G_3_|	n.s.
Cons	HS-LCL (G_1_)	288	27.37	7.35	16.8	0.00	0.04	| G_1_–G_2_|^∗∗^	0.36
	HRL-LCB (G_2_)	236	29.99	7.69				| G_2_–G_3_|^∗∗^	0.35
	HCB-RSLI (G_3_)	323	26.27	7.74				| G_1_–G_3_|	n.s.
Med	HS-LCL (G_1_)	288	26.68	8.16	22.78	0.00	0.05	| G_1_–G_2_|^∗∗^	0.42
	HRL-LCB (G_2_)	236	30.15	7.58				| G_2_–G_3_|^∗∗^	0.41
	HCB-RSLI (G_3_)	323	25.63	8.18				| G_1_–G_3_|	n.s.

*Cv, Convergence vs. divergence; Imp, Impulsiveness vs. reflectiveness; Ind, Field independence/dependence; Dem, Positive perception of how parents exert authority in the home; Per, Negative perception and expectations about social relations; Hos, Negative perception of the quality of acceptance and protection by parents; Obs, Difficulty in observing and retaining relevant information about social situations; Alt, Difficulty in finding alternative solutions for solving social problems; Cons, Difficulty in anticipating and understanding the consequences that could follow social behaviors; Med, Difficulty in choosing adequate means to the ends pursued by social behavior; HS-LCL(G_1_), high Stimulation-low Leadership and Conformity profile; HRL-LCB(G_2_), high Recognition and Leadership-low Conformity and Benevolence profile; HCB-RSLI (G_3_), high Conformity and Benevolence-low Stimulation and Recognition profile.*

Analysis of variance results show the existence of statistically significant differences in mean scores of the three groups in the following factors: *“Convergence vs. divergence”* [*F*_(2,844)_ = 6.18, *p* < 0.01, η^2^ = 0.01], *“Impulsiveness vs. reflectiveness”* [*F*_(2,844)_ = 19.79, *p* < 0.00, η^2^ = 0.05], *“Negative perception and expectations about social relations”* [*F*_(2,844)_ = 13.69, *p* < 0.00, η^2^ = 0.03], *“Negative perception of the quality of acceptance and protection by parents”* [*F*_(2,844)_ = 16.38, *p* < 0.00, η^2^ = 0.04], *“Difficulty in observing and retaining relevant information about social situations”* [*F*_(2,844)_ = 23.51, *p* < 0.00, η^2^ = 0.05], *“Difficulty in finding alternative solutions for solving social problems”* [*F*_(2,844)_ = 15.93, *p* < 0.00, η^2^ = 0.04], *“Difficulty in anticipating and understanding the consequences that could follow social behavior”* [*F*_(2,844)_ = 16.8, *p* < 0.00, η^2^ = 0.04], *“Difficulty in choosing adequate means to the ends pursued by social behavior”* [*F*_(2,844)_ = 22.78, *p* < 0.00, η^2^ = 0.05]. When *post hoc* comparisons are examined, the scores in the HRL-LCB Group are observed in all cases to be significantly higher than HS-LCL and HCB-RSLI, with a small effect size for these differences in *“Convergence vs. divergence”* (*HS-LCL* *d* = 0.22; *HCB-RSLI d* = 0.21), *“Impulsiveness vs. reflectiveness”* (*HS-LCL* *d* = 0.39; *HCB-RSLI d* = 0.38), *“Negative perception and expectations about social relations”* (*HS-LCL* *d* = 0.33; *HCB-RSLI d* = 0.32), *“Negative perception of the quality of acceptance and protection by parents”* (*HS-LCL* *d* = 0.36; *HCB-RSLI d* = 0.35), *“Difficulty in observing and retaining relevant information about social situations”* (*HS-LCL* *d* = 0.43; *HCB-RSLI d* = 0.42), *“Difficulty in finding alternative solutions for solving social problems”* (*AE-BCL* *d* = 0.35; *HCB-RSLI d* = 0.34), *“Difficulty in anticipating and understanding the consequences that could follow social behavior”* (*HS-LCL* *d* = 0.36; *HCB-RSLI d* = 0.35), *“Difficulty in choosing adequate means to the ends pursued by social behavior”* (*HS-LCL* *d* = 0.42; *HCB-RSLI d* = 0.41).

Finally, with respect to the “Field independence/dependence” factor, the ANOVA showed statistically significant differences in mean scores of the three groups [*F*_(2,844)_ = 22.74, *p* < 0.00, η^2^ = 0.05]. Examining the *post hoc* comparisons, the *HRL-LCB* Group shows significantly higher mean scores than the other two groups, *HS-LCL* or *HCB-RSLI*, with a small effect size (*d* = 0.42 and *d* = 0.41, respectively). In this case the Scheffe test also showed significant differences between the last two groups, *HS-LCL* and *HCB-RSLI,* showing that students with HS-LCL had a significantly higher mean score than the group of students with HCB-RSLI, again with a small effect size of these differences (*d* = 0.39).

## Discussion

The first specific goal of this study was to analyze the different combinations of interpersonal values and define the profiles characterized by the different levels of the five interpersonal values, for which the corresponding cluster analyses were done. Three different interpersonal value profiles were thus identified, each corresponding to different levels of student academic performance. We found one group was characterized by kindness, not exerting much authority and with little respect for the rules. A second group comprised of students who see themselves as recognized and admired by others, who exert authority, who do not respect the rules and are not very generous. This group, that is, subjects with low Benevolence and Conformity, show higher prevalence of failure and repeat the year more often. Thus the second specific goal of this study is met and the hypothesis that relates values like Benevolence, Conformity, and Stimulation with Academic Performance is reaffirmed (Ros et al., 1996).

Social competence, which is clearly a factor influencing the individual’s ability to become socially integrated (Mahar and Sullivan, 2002), is configured by cognitive attitudes and strategies (Trianes et al., 1997) and directly related to the individual’s values (Sutton and Smith, 1999), and thus the importance of this study which relates both. After analysis of the results, we were able to establish a profile of values that should be eliminated from the student, since they lead to attitudes inhibiting social relations and socially incompetent individuals. We should work on respecting rules, and achieving generous individuals who help others, while the values to be eliminated are Recognition and Leadership, that is, we should work to get students to keep from exerting authority and valuing admiration and recognition by others. It should be mentioned that in our study, Leadership is understood as negative, to the contrary of other studies where consideration for others is related positively to leadership and altruism (Cardozo et al., 2011). In this study, we also define the value profile leading to social thinking facilitating social relations and promoting socially competent individuals. Intervention programs should be carried out in the school or with the families themselves, to achieve high levels of kindness and consideration, respect for rules and generosity, and diminish the perception of recognition by others and exertion of authority.

It may be concluded that the common denominator of the two profiles, socially competent and incompetent, is Benevolence, that is, in the first profile we find generous individuals willing to help others, while in the second they are the opposite. So like other studies, and in agreement with the hypothesis posed, high levels of Benevolence are present in subjects with prosocial behavior (Fossati et al., 2012).

We cannot end without suggesting that many factors influence social thinking of individuals, and that in addition to values, perhaps other variables which influence the aspects of social thinking evaluated, such as attention, (Cluver and Gardner, 2006; Cao and Su, 2007), extroversion (Castaño and León del Barco, 2010), and aggressiveness (Fernández-Millán et al., 2002), etc., should be included in future studies. Nevertheless, the contribution of this study, by showing the values to be worked on and aspects to be avoided to improve Academic Performance of students and their social competence, is well worth mentioning.

## Conflict of Interest Statement

The authors declare that the research was conducted in the absence of any commercial or financial relationships that could be construed as a potential conflict of interest.
